# Effect of a Low-Dose/High-Frequency Training in Introducing a Nurse-Led Neonatal Advanced Life Support Service in a Referral Hospital in Ethiopia

**DOI:** 10.3389/fped.2021.777978

**Published:** 2021-11-25

**Authors:** Misrak Tadesse, Suzanne Hally, Sharla Rent, Phillip L. Platt, Thomas Eusterbrock, Wendmagegn Gezahegn, Tsinat Kifle, Stephanie Kukora, Louis D. Pollack

**Affiliations:** ^1^Wax & Gold Inc., Amarillo, TX, United States; ^2^Division of Neonatology, Department of Pediatrics, Johns Hopkins University School of Medicine, Baltimore, MD, United States; ^3^Division of Neonatology, Department of Pediatrics, Massachusetts General Hospital, Boston, MA, United States; ^4^School of Nursing, Endicott College, Boston, MA, United States; ^5^Division of Neonatology, Department of Pediatrics, University of Michigan, Ann Arbor, MI, United States; ^6^Pediatrix Medical Group, Department of Neonatology, Baptist St Anthony's Hospital, Amarillo, TX, United States; ^7^Division of Neonatology, Alta Bates Summit Medical Center, Berkeley, CA, United States; ^8^Saint Paul's Hospital Millennium Medical College, Addis Ababa, Ethiopia

**Keywords:** neonatal resuscitation, global health, neonatal mortality, Ethiopia, delivery room, low-and middleincome countries, low-dose high-frequency training, quality improvement

## Abstract

**Background and Objective:** In Ethiopia, birth asphyxia causes ~30% of all neonatal deaths and 11–31% of deaths among neonates delivered in healthcare facilities that have breathing difficulty at birth. This study aimed to examine the impact of low-dose, high-frequency (LDHF) training for introducing a nurse-led neonatal advanced life support (NALS) service in a tertiary care hospital in Ethiopia.

**Methods:** Through a retrospective cohort study, a total of 12,001 neonates born post-implementation of the NALS service (between June 2017 and March 2019) were compared to 2,066 neonates born before its implementation (between June 2016 and September 2016). Based on when the neonates were born, they were divided into six groups (groups A to F). All deliveries occurred in the inpatient Labor and Delivery Unit (LDU) at St. Paul's Hospital Millennium Medical College. The number of neonatal deaths in the LDU, neonatal intensive care unit (NICU) admission rate, and proportion of neonates with normal axillary temperature (36.5–37.5°C) within the first hour of life were evaluated. Data were analyzed using the χ^2^ test, and *p*-values < 0.05 were considered statistically significant. Following the implementation of the NALS service, semi-structured interviews with key stakeholders were conducted to evaluate their perception of the service; the interviews were recorded, transcribed, and coded for thematic analysis.

**Results:** There was a decrease in the proportion of neonates who died in the LDU (from 3.5 to 1%) during the immediate post-implementation period, followed by a sustained decrease over the study period (*p* < 0.001). The change in the NICU admission rate (from 22.8 to 21.2%) was insignificant (*p* = 0.6) during this initial period. However, this was followed by a significant sustained decrease (7.8% in group E and 9.8% in group F, *p* < 0.001). The proportion of newborns with normal axillary temperature improved from 46.2% during the initial post-implementation period to 87.8% (*p* < 0.01); this proportion further increased to 99.8%. The program was perceived positively by NALS team members, NICU care providers, and hospital administrators.

**Conclusion:** In resource-limited settings, LDHF training for neonatal resuscitation improves the neonatal resuscitation skills and management of delivery room attendants.

## Introduction

Neonatal mortality, defined as death that occurs within the first 28 days of life, accounts for 47% of global deaths of children under the age of 5 years (1). Most neonatal deaths occur due to preventable conditions, such as birth asphyxia. Approximately one-third of such deaths occur within the first day of life, and ~3-quarters of such deaths occur within the first week of life (1). Neonatal deaths in Sub-Saharan African (SSA) countries account for 37% of under-five mortality (2). In 2019, Ethiopia ranked second in Africa and fourth in the world among the top 10 countries with the highest number of neonatal deaths, with an estimated 99,000 neonatal deaths (1, 3). Between 2011 and 2019, significant strides toward the improvement of maternal healthcare have been made in Ethiopia. With these improvements, the proportion of women receiving antenatal care increased from 19 to 43%, and the proportion of births that occurred in health facilities increased from 10 to 48% (4). However, the neonatal mortality rate (NMR) remains high at 33 deaths per 1,000 live births, a minimal decrease was observed from 37 deaths per 1,000 live births in 2011 (4).

The urgency associated with neonatal mortality has increased since even newborns who benefit from being born in healthcare facilities are not faring well (5–7). In Ethiopia, birth asphyxia is the cause of ~30% of all neonatal deaths and 11–31% of neonatal deaths among those who were delivered in healthcare facilities and required assistance for breathing difficulties (8–10).

There are several factors behind the high NMR, including the fact that only an estimated 50% of births are attended by skilled providers, and there is a shortage of providers skilled in the provision of neonatal resuscitation and postnatal care (4, 9, 11, 12). In high-income countries, the standardization of training, knowledge, and skills for neonatal resuscitation has resulted in a significant decrease in neonatal mortality (13). Although neonatal outcomes in low- and middleincome countries (LMIC) could be improved through the implementation of such programs, progress in this regard has been hindered by challenges associated with the provision of supplies, education, and training (14). Only 15% of hospitals in SSA countries have the capability for basic neonatal resuscitation (15). The analysis from the 2016 national Emergency Obstetric and Newborn Care Survey in Ethiopia is in line with this finding (12). Inadequately trained staff, missing equipment, poor skill retention, and poor staffing are some of the barriers to the availability of basic resuscitation capabilities (16).

While the low-dose, high-frequency (LDHF) training model has been endorsed to foster skill retention following initial training, building a sustainable capacity for the improvement of the quality of neonatal resuscitation in an LMIC setting will ultimately depend on the development and implementation of programs involving the education and mentoring of clinicians for the creation of a train-the-trainer model (13, 17–19).

The neonatal advanced life support (NALS) program implemented at St. Paul's Hospital Millennium Medical College (SPHMMC) builds on the Helping Babies Survive programs designed by the American Academy of Pediatrics and includes advanced neonatal resuscitation, post-resuscitation care with respiratory and cardiovascular support, intra- and inter-facility transportation of sick newborns, data collection, and quality-improvement methodologies. As described by Jhpiego, an LDHF training model was utilized that focused on short and frequent didactic sessions that helped build competency through simulation and case-based learning. It was team-focused and facility-based, and learners had ongoing exposure to content after initial training followed by intermittent supportive mentorship and performance evaluation spaced over 12 months (19).

## Materials and Methods

### Design

This retrospective cohort study was conducted in the inpatient Labor and Delivery Unit (LDU) at SPHMMC, a regional tertiary referral hospital in < city> Addis Ababa </city>, Ethiopia, that serves a catchment population of ~5 million individuals. Annually, more than 12,000 deliveries are performed at SPHMMC. Approximately, two-thirds of the newborns are delivered in the inpatient LDU while the remaining are delivered in the outpatient LDU.

### Intervention

In 2015, SPHMMC invited Wax and Gold, Inc., a US-based 501(c)3 non-profit organization working in Ethiopia in education and capacity building to improve newborn healthcare, to establish a collaborative partnership for the design and implementation of a NALS service. After interviews with key stakeholders and the performance of a needs assessment, a 12-month longitudinal curriculum including didactic lectures, simulation scenarios, mentored clinical practicum, case reviews, and quality-improvement training was developed to train a dedicated team of nurses to attend all high-risk deliveries and provide newborn stabilization and resuscitation. The NALS team was selected from existing hospital staff, and it included 10 neonatal intensive care unit (NICU) nurses and one midwife, all of whom had Bachelor of Science degrees and at least 2 years of experience with bedside patient care.

**Figure 1:**
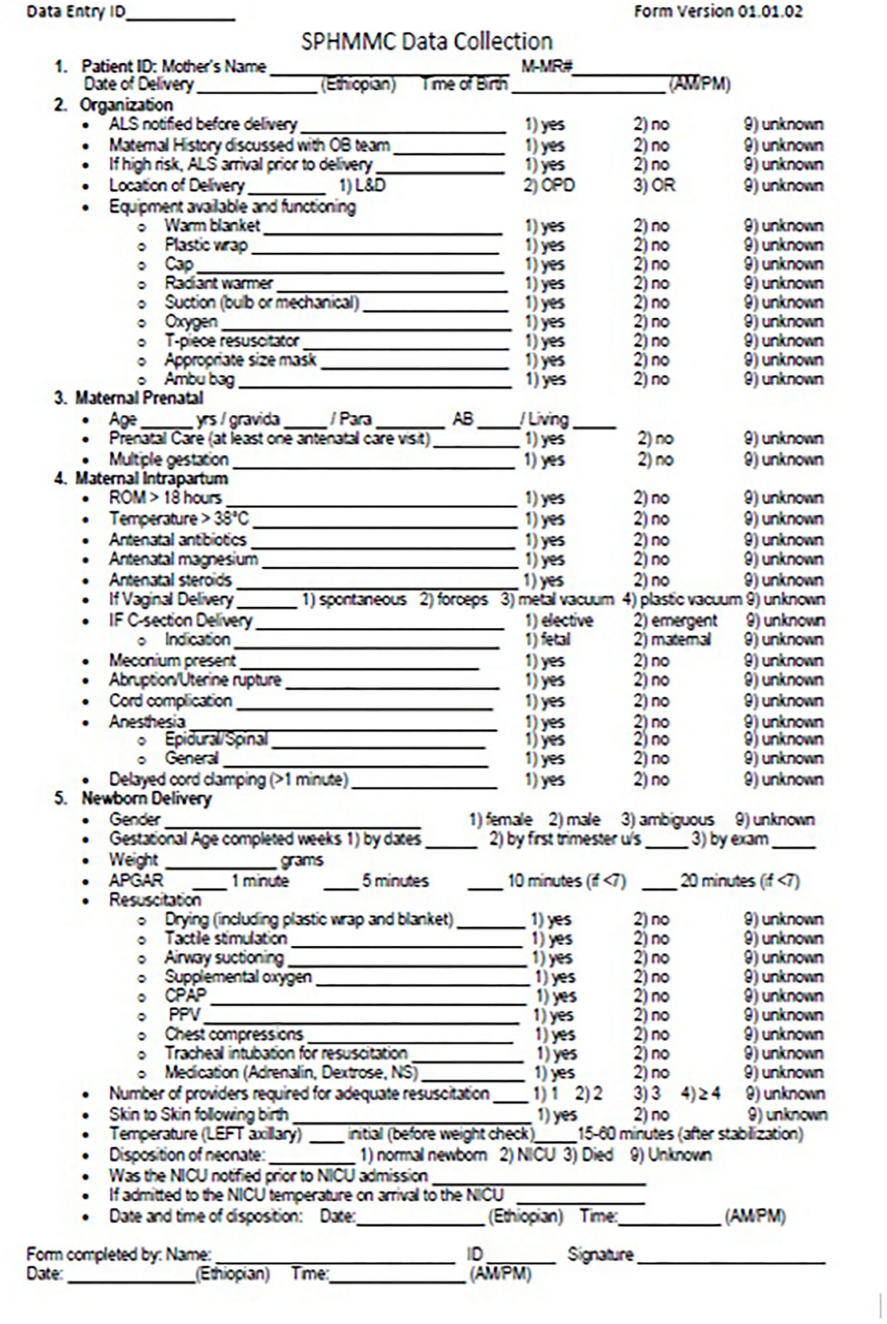
Standardized data collection form.

**Figure 2:**
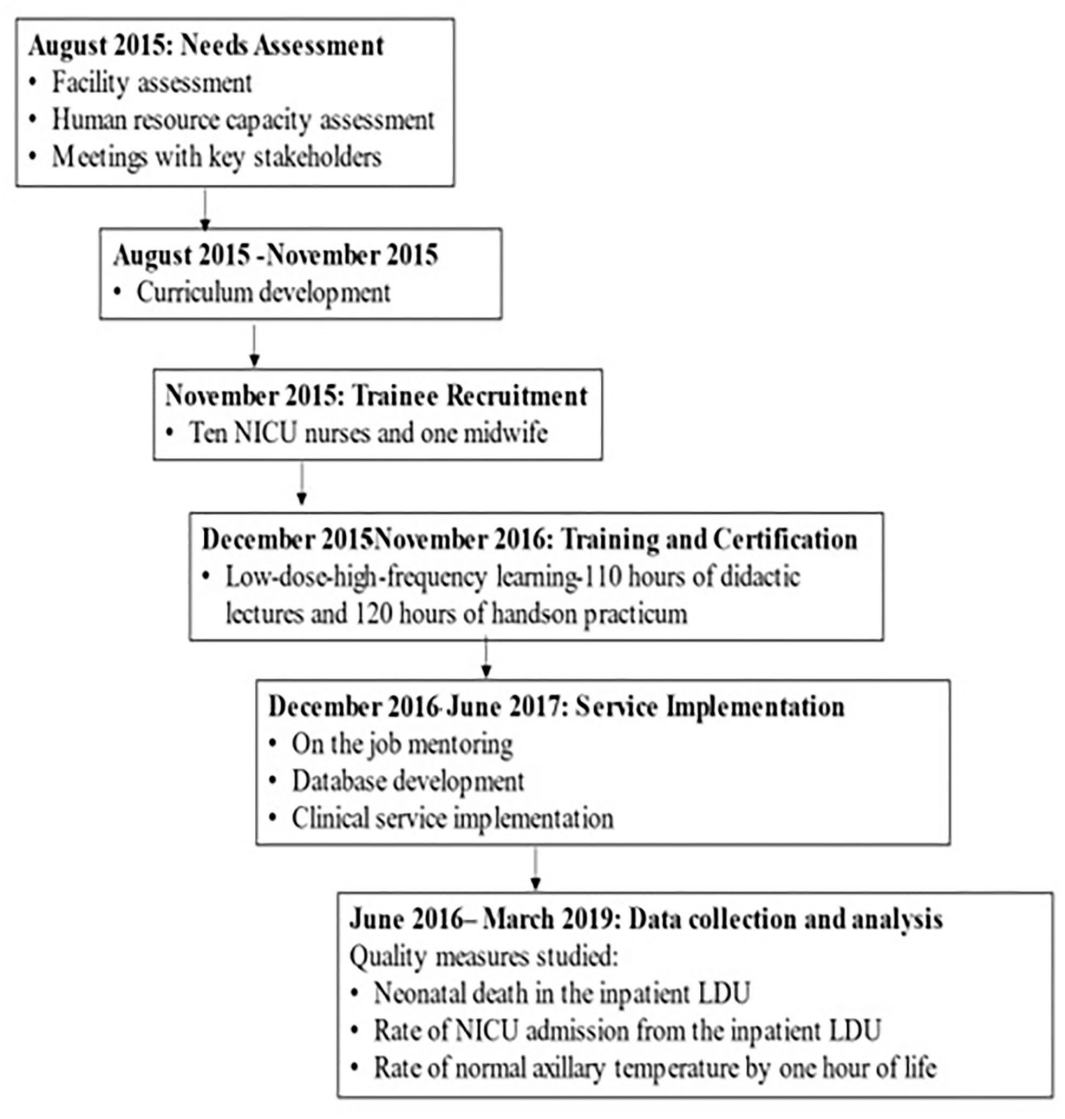
Timeline of project design, implementation, and study.

The training curriculum was developed using existing educational platforms, including the American Academy of Pediatrics' Helping Babies Survive and Neonatal Resuscitation programs, the Sugar, Temperature, Airway, Blood Pressure, Lab work, and Emotional support (S.T.A.B.L.E) program, and the comprehensive competency-based orientation program designed by the Association of Women's Health, Obstetric, and Neonatal Nurses (Table 1) (20–23). As part of the interventions, a plastic wrap was used for thermoregulation, and T-piece resuscitators were used to provide positive pressure ventilation and continuous positive airway pressure. The training involved 110 h of didactic presentations and simulation-based training followed by 120 h of hands-on practicum in the LDU with case review, 20 h of supplemental lectures and interactive learning regarding quality-improvement methodologies and data management, and 6 additional months of intermittent bedside clinical mentorship. Experts in neonatal care and resuscitation from the US and Ethiopia led the didactic and hands-on portions of this training. Concurrently, a standard data collection form was developed (Figure 1) and validated for user-friendliness, workflow, and acceptance. Using MySQL Workbench (Oracle Corporation, Austin, TX, USA), the Information Technology Department of SPHMMC built and maintained a computerized database that was obtained using the data-collection form (24). The NALS service was initially rolled out in the inpatient LDU in December 2016 and was fully implemented in June 2017 (Figure 2).

**Table 1:** NALS curriculum developed to train a dedicated staff to provide newborn stabilization and resuscitation.

**NALS Curriculum**		**Hours inTraining**
Maternal, fetal and newborn pathophysiology	Didactic teaching developed by WAG, focusing on a thorough understanding of normal and pathologic maternal, fetal, and newborn physiology.	Total 30
Helping babies survive	Didactic and simulation training adapted from AAP Helping Babies Breathe (HBB): initial steps of neonatal resuscitation for care in The Golden Minute including drying, suctioning, stimulation, and positive pressure ventilation; Essential Care for Every Baby (ECEB): newborn care practices from delivery to hospital discharge; and Essential Care for Small Babies (ECSB): newborn care specialized for small and premature infants.	Total 20
S.T.A.B.L.E (S, Sugar and Safe care T, Temperature A, Airway B, Blood pressure L, Lab E, Emotional support)	Didactic teaching adapted from S.T.A.B.L.E. neonatal nursing education curriculum on topics of evaluation and management of newborns, including glucose monitoring and management, assessment of respiratory status and blood pressure, laboratory evaluation, and parental support.	Total 30
Neonatal resuscitation program	Didactic and simulation training including low fidelity simulation scenarios based on the AAP evidence-based approach to neonatal resuscitation and team-based care of the newborn at delivery. This included advanced airway techniques including intubation, cardiopulmonary resuscitation (CPR) as	Total 30
	well as umbilical catheterization and administration of epinephrine.	
One to one practicum in the delivery room	Supervised ALS trained provider attendance of deliveries at SPHMMC by WAG volunteers (neonatologists, neonatal nurse practitioners, and neonatal nurses from the US and Canada) with continued hands-on training and mentoring.	120
Data collection/quality improvement	Didactic teaching on outcome improvement through continuous analysis of practice performance and modification, as well as hands-on teaching on the topic of data collection using the developed form and electronic data entry.	Total 20

*The curriculum was taught over a 12-month period to the NALS team (10 NICU nurses and 1 midwife) selected from existing hospital staff*.

### Data Collection and Analysis

Three outcomes were studied: neonatal death in the inpatient LDU, rate of admission to the NICU from the inpatient LDU, and the proportions of neonates with normal axillary temperature (36.5–37.5°C) within 1 h of life. After the implementation of the NALS program, five separate groups (groups B to F) consisting of all newborns from the inpatient LDU were identified and compared to a group of neonates (group A) born before the implementation of the program (between June 4, 2016 and September 17, 2016) from the same LDU. Data were entered in realtime on a paper form and then transferred to an electronic database maintained in a password-protected desktop computer in the LDU. Before the introduction of the NALS program, neonatal temperatures were not recorded.

Initial post-implementation data were collected from June 4, 2017 to September 17, 2017 (group B). There was a subsequent 2-month pause for the validation of data entry of group B and additional training. The second data entry period extended from November 15, 2017 to May 24, 2018 (group C); again, this was followed by validation. Starting from July 1, 2018, data were collected and analyzed quarterly (groups D, E, and F). An evaluation was done after each quarter to ensure appropriate data collection and to look for any negative trends in the outcome measure. Data were analyzed using the χ^2^ test, and *p* < 0.05 was considered statistically significant.

Additionally, a qualitative analysis of the NALS program was performed by an independent study team that identified staff perceptions of the feasibility, effectiveness, and acceptability of the NALS program. Semi-structured interviews were conducted at SPHMMC in January 2018. Twenty individuals, including NALS team members, NICU nurses, physicians, and hospital administrators, were interviewed to assess the perceived impact of the introduction of the NALS program on neonatal outcomes. For this purpose, medical staff who were willing to participate were identified ahead of time by the lead SPHMMC research partner. Participants were then recruited based on a convenience sample on the day of the interview. Interviews were conducted in English with the assistance of a local interpreter when needed. All interviews were audio-recorded, transcribed verbatim, and subsequently underwent thematic analysis. This study was approved by the institutional review board and leadership of SPHMMC. No parental consent was required for this quality improvement initiative. Consent was obtained from the healthcare providers participating in the survey.

## Results

**Figure 3:**
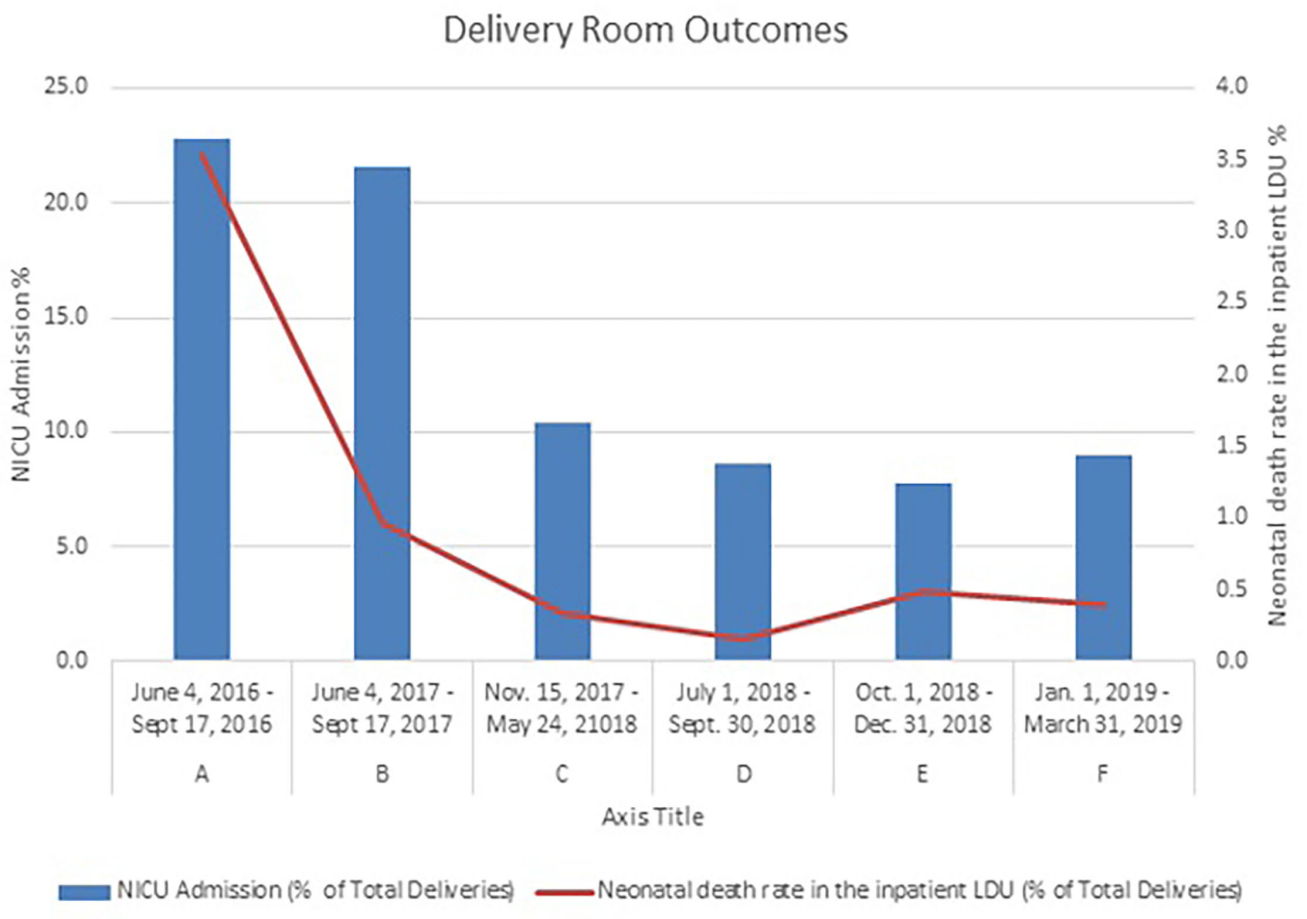
Neonatal death rate in the inpatient LDU and NICU admission rate before and after the implementation of the NALS service. Both fell significantly between groups A and C (p < 0.001) and were sustained further between groups D and F.

During the initial period of the analysis of data on group A (preimplementation) and groups B and C (post-implementation), the number of deliveries remained stable, with a mean of 19.7, 21.9, and 17.1 deliveries per day in groups A, B, and C, respectively (Table 2). Concurrently, the rate of NICU admissions from the inpatient LDU decreased significantly from 22.8% in group A to 10.4% in group C (*p* < 0.001). After the implementation of the NALS program, there was a decrease in the proportion of neonates who died (neonatal mortality) in the inpatient LDU; neonatal mortality in the inpatient LDU was 3.5, 1.0, and 0.3% in groups A, B, and C, respectively (*p* < 0.001) (Table 3; Figure 3). The NALS providers were not responsible for assigning the cause of death. Only the occurrence of death was recorded in the NALS database. The target axillary temperatures (36.5–37.5°C) were achieved within the first h of life in 46.2% of the neonates in group B and 87.8% (*p* < 0.01) of the neonates in group C (Table 4).

**Table 2:** Birth rate and NICU admission rate before and during the initial two periods after implementation of the NALS service.

**Cohort**	**Dates**	**Deliveries (*n*)**	**Deliveries per day, mean**	**NICU admission (%)**	**NICU admissions per day, mean**
A	June 4, 2016–Sept 17, 2016	2,066	19.7	472 (22.8%)	4.5
B	June 4, 2017–Sept 17, 2017	2,299	21.9	496 (21.6%)	4.7
C	Nov 15, 2017–May 24, 2018	4,269	17.1	445 (10.4%)	1.8
D	July 1, 2018–Sept. 30, 2018	1,239	13.6	106 (8.6%)	1.2
E	Oct. 1, 2018–Dec. 31, 2018	1,205	13.2	94 (7.8%)	1.0
F	Jan 1, 2019–March 31, 2019	2,989	33.2	270 (9.0%)	3.0

*The NICU admission rate is expressed as a proportion of neonates delivered at SPHMMC inpatient LDU who required admission to the NICU. NICU admission rates fell significantly between Groups A and C (p < 0.001)*.

**Table 3:** Neonatal death rate in the inpatient LDU before and after the implementation of the NALS service.

**Cohort**	**Dates**	**Deliveries (*n*)**	**Neonatal death rate in the inpatient LDU *n* (%)**
A	June 4, 2016–Sept 17, 2016	2,066	73 (3.53%)
B	June 4, 2017–Sept 17, 2017	2,299	22 (0.96%)
C	Nov 15, 2017–May 24, 2018	4,269	14 (0.33%)
D	July 1, 2018–Sept. 30, 2018	1,239	2 (0.16%)
E	Oct. 1, 2018–Dec. 31, 2018	1,205	6 (0.49%)
F	Jan 1, 2019–March 31, 2019	2,989	12 (0.4%)

*Mortality rate decreased significantly between groups A and C (p < 0.001) and remained stable between groups C–F (p = 0.89)*.

**Table 4:** Rate of axillary temperature in the normal range (36.5–37.5°C) after the implementation of the NALS service.

**Cohort**	**Dates**	**Deliveries (*n*)**	**Axillary temperatures between 36.5–37.5^°^C in the first h of life (%)**
A	June 4, 2016–Sept 17, 2016	2,066	Not measured
B	June 4, 2016–Sept 17, 2017	2,299	1,062 (46.2%)
C	Nov 15, 2017–May 24, 2018	4,269	3,748 (87.8%)
G	July 1, 2018–March 31, 2019	5,433	5,420 (99.8%)

*No data were collected before the NALS implementation. Rates of normothermia improved significantly between Groups B and C (p < 0.01). Group G represents a composite of data collected following cohort C*.

Over the study period, in groups D and E, the number of deliveries per day decreased to 13.6 and 13.2, respectively (Table 2). No specific reasons were identified for the decline in the number of births. There was a significant increase in the number of deliveries per day in group F to 33.2, attributed to a process change implemented to consolidate all the deliveries into the inpatient LDU (Table 2).

During the same time, the decrease in the NICU admission rate observed in group C remained stable; among groups D, E, and F, the NICU admission rate ranged from 7.8 to 9% (*p* = 0.11) (Table 2; Figure 3). A sustained decrease in mortality was also observed among groups D, E, and F, ranging from 0.2–0.5% (*p* = 0.89) (Table 4; Figure 3). Likewise, there was a sustained improvement in the percentage of neonates who achieved target axillary temperatures within the first h of life; 99.8% of patients in group G, the combined cohort representing groups D to F, achieved axillary temperatures within the target temperature range (Table 4). There were no worsening of outcomes identified at any checkpoint that required a review of the NALS process.

In the subsequent qualitative analysis, the overall impact of the implementation of the NALS program on neonatal outcomes was evaluated. The responses of the NALS team members and those of the NICU care providers and hospital administrators were analyzed separately. The themes identified in NALS team-member interviews included the value of education and training, pride in work, and confidence in skills. Themes identified in the interviews of the NICU staff and hospital officials included praise for program outcomes, improvement in workflow, and desire for additional training opportunities. All interviewees were pleased with the progress in neonatal outcomes observed in SPHMMC following the implementation of the NALS service. Illustrative quotes from NALS team members have been included in Table 5, and quotes from the NICU providers and senior hospital administrators have been included in Table 6.

**Table 5:** Quotes regarding the impact made by the Advanced Life Support (ALS) Team at St. Paul's Hospital.

**Major Themes with Representative Quotes from Neonatal ALS Team Members**
**Value of NALS Education and Training**
“We were taught to take care of the babies very well. Resuscitate. We have enough equipment, manpower … **our team is equipped by materials and education**. I think this is it. That is the reason for improvements in outcomes.” –NALS Team Nurse
**Confidence in Skills**
“Our role is that we care for newborns if they need resuscitation. **If the baby is meconium aspirated, or preterm, or struggling to breathe, and any other baby who needs our help we give it**. We have received specialized training and skills training, so I believe we have made an impact. We have impacted outcomes and babies are surviving because they are receiving resuscitation.” –NALS Team Nurse
“Outcomes are much improved. **The change is from the way resuscitation is done**. How to resuscitate the babies immediately, with a **focus on the golden time of resuscitation**. Immediately when the baby comes, if the baby cries already, we dry the baby and wrap with a dry towel and keep him warm. If he is not crying, we immediately see the airway … whether it is clear or not. We clear the airway, wrap and stimulate the baby. If he is not crying, not improving still, will do MR SOPA. This … after MR SOPA, most of the babies will cry and give a response. **That is how we know we have done well**.” –NALS Team Nurse
**Pride in Work**
“At this hospital, there is a special team called the ALS team. In other hospitals the babies are handled with midwives. These midwives are giving newborn babies resuscitation, but it is not as good. But this training, ALS team training, gives us special resuscitation for newborn babies. This is good. **It decreases neonatal mortality**; especially immediate neonatal deaths have decreased. **I think we can be proud of this change**.” –NALS Team Nurse
“I used to work in the NICU and would see babies referred from the obstetrical ward being hypothermic, hypoglycemic, not being resuscitated. We were the ones doing the resuscitation here. **Since we can now get to them early and doing the resuscitation there, I feel really good**.” –NALS Team Nurse

*Quotes were obtained during the qualitative interviews of NALS Team Members in January 2018*.

**Table 6:** Quotes regarding the impact made by the Advanced Life Support (ALS) Team at St. Paul's Hospital.

**Major Themes with Representative Quotes from Non-NALS Neonatal Care Providers**
**Praise for program outcomes**
“The ALS team manages our resuscitations. They are the ones who resuscitate on the way up to here. They have received special training about resuscitating. We have seen a big difference because of them. **More babies are surviving initially and later on**.” –Neonatal Nurse
“There is a new program in place for resuscitation. They have trained some special nurses, I think ones that used to be in the NICU, to be the managers of resuscitating babies. From what I hear and what I have seen, **they do a good job, especially when you consider the need to keep babies warm and manage birth asphyxia, which is a real problem here**.” – PediatricResident
“The ALS team is doing well; they are doing good with respect to managing hypothermia. Usually, the hypothermia upon arrival is present if they are admitted from outside. Usually these babies were arriving with hypothermia. But **in those that have delivered in St. Paul, the hypothermia rate is low**. It is a lot less common now because of the **attention paid at delivery**.” – Pediatric Resident
**Improvement in workflow**
“The ALS nurses are also deciding if the baby needs to come to the NICU by assessing the condition after birth. For these deliveries, **survival is better**. Also, **workload is better in the NICU** because the ALS team has already resuscitated most of the time.” – Neonatal Fellow
**Desire for additional training opportunities**
“To help the newborn survival rate we have a new program called ‘Advanced Life Support' for the newborns. These nurses have significantly **decreased the number of admissions to the NICU**. And we have seen a significant drop in early newborn deaths in the labor ward. They provide immediate and dedicated care to the newborn, and the outcomes have been impressive. So, **whatever we did with the nurses, the resuscitation training in the labor ward, we have to replicate it in the NICU**.” – Senior Hospital Official Executive
“The ALS nurses are trained on resuscitation, but the nurses in the **NICU need similar training**. With the ALS nurses we have seen significant improvement in early neonatal deaths and on the number of babies being admitted for NICU, especially attributed to birth asphyxia.
“These **outcomes are due to training, and retraining**. Success comes from obtaining skills, but also, those skills need to be maintained.” – Senior Pediatrician

*Quotes were obtained during qualitative interviews of neonatal nurses and physicians in January 2018*.

## Discussion

In this study, we quantified the improvement in immediate postnatal outcomes following the provision of education and skills training; the study findings demonstrated the feasibility of teaching and sustaining advanced neonatal-resuscitation skills to healthcare providers in an LMIC setting. SPHMMC is the only public hospital in Ethiopia with certified NALS providers responsible for all high-risk deliveries, and our study is the first one in Ethiopia to document that LDHF training with a curriculum developed using existing programs with proven effectivity, coupled with extensive, consistent, and repetitive theoretical and hands-on bedside training in similar clinical-practice environments, is effective. This training program could serve as a model for the dissemination of neonatal-resuscitation skills in other healthcare institutions and hospitals in LMICs.

For the implementation of this training model, we relied on buy-in from key stakeholders to create an efficient and functional NALS program. Similar to almost all hospitals in Ethiopia, SPHMMC, despite being the largest delivery service in the country, did not have healthcare providers adequately trained in neonatal resuscitation. The administration of SPHMMC envisioned training and developing a NALS service in the delivery room. The engagement of stakeholders in pediatrics, obstetrics, nursing, and information technology was a crucial aspect of the collaboration and innovation required for the implementation and maintenance of this program.

In addition to the LDHF training strategy, the fact that the pace of the training was set according to that of the slowest learner, and the training was conducted in the same setting where the NALS team would eventually practice contributed significantly to the success of the project. The NALS team members were able to identify challenges in their practice environment and work on solutions. Intermittent mentoring in the inpatient LDU carried out by Wax and Gold physicians and nursing volunteers for the first 6 months following the implementation of the service helped the team reinforce their skills and build their confidence.

An additional feature of this NALS program was the emphasis on nurse-driven education and sustainability efforts. Job dissatisfaction leading to high attrition rates among nursing personnel is a major problem in the Ethiopian public healthcare system (25). Furthermore, inconsistent training in neonatal resuscitation among caregivers is a major contributor to persistently high neonatal mortality and morbidity rates in Ethiopian referral hospitals (26). The NALS training at SPHMMC has equipped these providers with the skills and knowledge required for them to be confident and satisfied with their abilities, resulting in a dramatic improvement in neonatal mortality and morbidity immediately after birth. These objective outcome improvements have led the NALS team to be recognized as a vital element of neonatal care at SPHMMC.

The continued success of this model relies on the persistent utilization of neonatal resuscitation skills by trained providers and the eventual training of others by those clinicians. Members of the first NALS cohort who expressed a desire to teach others, in addition to demonstrating exceptional clinical skills were invited to take a leadership role in training the second cohort to develop a self-sustaining program with the support of the hospital leadership. Two classes of NALS providers have been certified to date.

Following the successful implementation of the NALS program at SPHMMC, leadership at SPHMMC and Wax and Gold have initiated formal collaboration with the Ministry of Health and the Addis Ababa Regional Health Bureau to pilot the NALS service in health centers and non-referral hospitals in Addis Ababa.

A key strength of this study was the large number of neonates delivered at SPHMMC, which allowed for the inclusion of large cohort sizes with relatively stable demographics. The only change associated with a clinical practice that occurred during the study period was the introduction of the NALS service. Furthermore, a group of 11 initial NALS providers began training together and remained in their positions throughout the study period and six more NALS providers from the second cohort joined the team in September 2018. The NALS providers were present as a supplement to the standard staffing protocol in LDU. Additionally, data were captured in realtime, electronically entered, and verified by a third party. Finally, the semi-structured interviews used in the qualitative analysis were carried out by researchers not involved in the design or education of the NALS team; thus, a bias that could have been introduced by the interviewer or interviewees was avoided.

We encountered several challenges that have been well-described in previous studies addressing the implementation of neonatal resuscitation education in LMICs (27–30). Resistance to change was vocalized by nurses and midwives working in the delivery room who were concerned about the loss of relevance and job security. Staff concerns were addressed through a series of group meetings during which outcome data were presented, discussed, and used to support the rationale and intent of implementing this new clinical service. Process issues, including a lack of functioning basic equipment and availability of consumable products, were noted. This issue was initially managed using supplies for clinical service and instruction that were donated by Wax and Gold; ultimately, SPHMMC's Central Supply and Purchasing Department was able to identify and secure the necessary equipment and inventory.

Limitations of our study include incomplete data on demographic characteristics of the newborns both in the preimplementation period and in groups B and C, causes of death were not recorded, and temperatures in newborns within the first h of life were not recorded before the NALS service rollout. Although no other QI projects were on going in the LDU during the NALS service implementation period, further research is necessary to identify if there was a direct correlation between the improved outcome indicators and the implementation of the NALS service. With respect to the qualitative analysis, the use of an interpreter in certain interviews is a notable limitation of this study because some nuances may have been lost; however, allowing respondents to communicate in their language might also have led to the obtainment of responses that were more thorough than the responses that would be obtained if their native language had not been used. Lastly, as with any interview-based project, there is a possibility of responses being biased due to a desire to avoid presenting a negative opinion of the program to a visiting physician.

## Conclusion

Our study demonstrates the efficacy and acceptability of LDHF training to implement a NALS service at a tertiary care hospital in Ethiopia. Through the partnership, capacity-building, and focused quality-improvement methods, the implementation of the NALS program led to significant and sustained improvements in the neonatal resuscitation and management skills of delivery room attendants. This may have contributed to the reduction in the observed neonatal mortality and morbidity. Additionally, the qualitative analysis revealed the key stakeholders' buy-in and support of the NALS program. With respect to the goal of improving neonatal outcomes in LMIC with the highest neonatal mortality and morbidity rates, continued collaboration with local providers and key administrative and government stakeholders are vital to the development, implementation, and scale-up of educational programs for neonatal-resuscitation providers.

## Data Availability Statement

The raw data supporting the conclusions of this article will be made available by the authors, without undue reservation.

## Ethics Statement

The studies involving human participants were reviewed and approved by Institutional Review Board (IRB) of St. Paul's Hospital Millennium Medical College. Written informed consent for participation was not required for this study in accordance with the national legislation and the institutional requirements.

## Author Contributions

LP, MT, PP, SH, SR, SK, TE, and WG conceptualized and designed the study. MT, PP, SH, TE, and TK collected the data, while MT and SR analyzed the data. LP, MT, and SH drafted the initial manuscript. All authors contributed to the article and approved the submitted version.

## Conflict of Interest

The authors declare that the research was conducted in the absence of any commercial or financial relationships that could be construed as potential conflicts of interest. Wax and Gold, Inc. is a 501(c)3 non-profit volunteer-based organization working in Ethiopia in education and capacity building to improve newborn healthcare. It has no paid staff, and all its members and founders are volunteers.

## Publisher's Note

All claims expressed in this article are solely those of the authors and do not necessarily represent those of their affiliated organizations, or those of the publisher, the editors and the reviewers. Any product that may be evaluated in this article, or claim that may be made by its manufacturer, is not guaranteed or endorsed by the publisher.

## Abbreviations

LDHF: low-dose, high-frequency
LDU: Labor and Delivery Unit
LMIC: low- and middle-income countries
NALS: neonatal advanced life support
NICU: neonatal intensive care unit
NMR: neonatal mortality rate
SPHMMC: St.Paul's Hospital Millennium Medical College
SSA: Sub-Saharan African
S.T.A.B.L.E: Sugar, Temperature, Airway, Lab work, and Emotional support.
